# Gallic Acid Promotes Wound Healing in Normal and Hyperglucidic Conditions

**DOI:** 10.3390/molecules21070899

**Published:** 2016-07-08

**Authors:** Dong Joo Yang, Sang Hyun Moh, Dong Hwee Son, Seunghoon You, Ann W. Kinyua, Chang Mann Ko, Miyoung Song, Jinhee Yeo, Yun-Hee Choi, Ki Woo Kim

**Affiliations:** 1 Department of Pharmacology, Wonju College of Medicine, Yonsei University, Wonju 26426, Korea; ydj1991@yonsei.ac.kr (D.J.Y.); donghweeson@gmail.com (D.H.S.); bloodship90@naver.com (S.Y.); annmendi@gmail.com (A.W.K.); changmko@yonsei.ac.kr (C.M.K.); 2 Department of Global Medical Science, Wonju College of Medicine, Yonsei University, Wonju 26426, Korea; 3 Anti-Aging Research Institute of BIO-FD & C Co. Ltd., Incheon 21990, Korea; biofdnc@gmail.com (S.H.M.); mysong@biofdnc.com (M.S.); 4 Department of Wellness & Healthy Aging, Wonju College of Medicine, Yonsei University, Wonju 26426, Korea; 5 Jeongseon Agricultural Extension Center, Jeongseon 26103, Korea; 1257@korea.kr

**Keywords:** gallic acid, wound healing, cell migration, hyperglucidic condition

## Abstract

Skin is the outermost layer of the human body that is constantly exposed to environmental stressors, such as UV radiation and toxic chemicals, and is susceptible to mechanical wounding and injury. The ability of the skin to repair injuries is paramount for survival and it is disrupted in a spectrum of disorders leading to skin pathologies. Diabetic patients often suffer from chronic, impaired wound healing, which facilitate bacterial infections and necessitate amputation. Here, we studied the effects of gallic acid (GA, 3,4,5-trihydroxybenzoic acid; a plant-derived polyphenolic compound) on would healing in normal and hyperglucidic conditions, to mimic diabetes, in human keratinocytes and fibroblasts. Our study reveals that GA is a potential antioxidant that directly upregulates the expression of antioxidant genes. In addition, GA accelerated cell migration of keratinocytes and fibroblasts in both normal and hyperglucidic conditions. Further, GA treatment activated factors known to be hallmarks of wound healing, such as focal adhesion kinases (FAK), c-Jun N-terminal kinases (JNK), and extracellular signal-regulated kinases (Erk), underpinning the beneficial role of GA in wound repair. Therefore, our results demonstrate that GA might be a viable wound healing agent and a potential intervention to treat wounds resulting from metabolic complications.

## 1. Introduction

Gallic acid (GA) is a 3,4,5-trihydoxybenzoic acid, a phenolic acid found in almost all plants including fruits, leaves, and wild flowers [1,2], and has been reported to possess powerful health benefits, such as antioxidant, anti-inflammatory, analgesic, neuroprotective, anticancer, and anti-diabetic properties [3].

The skin comprising of outer epidermis, underlying connective tissue, and dermis functions as a barrier that protects the body from environmental stressors, such as pathogens, excessive water loss, temperature, and physical stress [4]. Skin injuries, such as abrasions and incisions, may cause damage to the epidermis and/or dermal layers, necessitating repair through a wound healing process comprised of four sequential phases; homeostasis, inflammation, proliferation/re-epithelialization, and maturation [5]. When this well-ordered process is disrupted by factors, such as age, hyperglycemia, poor circulation, repeated trauma, continuous pressure, infections, or systemic illnesses, the wound fails to close in an expected time frame developing into a chronic wound [6]. Both acute and chronic wounds represent a major public healthcare burden affecting a relatively large population [7]. Proper wound healing is essential not only for the restoration of disrupted anatomical continuity and the barrier function of the skin, but also for reducing the risk of infection and further complications [5].

The use of natural products from plants for treatment of wounds has been practiced since ancient times. Due to the fact that plant-extracted treatments are easily accessible and relatively safe, there is a steadily growing interest in using natural compounds to prevent and combat skin pathologies [8]. Moreover, polyphenols have been widely used in traditional medicines to treat several chronic skin diseases, such as psoriasis and vitiligo, and they are also known to be therapeutically beneficial in wound healing and show anti-inflammatory effects when applied topically [9,10]. Despite the fact that GA exhibits several beneficial properties including antioxidant, anti-inflammatory, and anti-tumor activities, there is only little evidence on the role of GA in skin and skin pathology. Here, we studied the effect of GA on would healing in human keratinocytes. Previously, our group studied the beneficial effects of GA on metabolic stress conditions and found improvement in whole body metabolism by GA treatment [11]. In addition, GA has shown strong protective effects on disease progression in type 1- and type 2-diabetes animal models [12]. Diabetes mellitus (DM) is characterized by delayed and impaired skin wound healing, leading to chronic ulcers, a common complication of DM [13]. We examined the effect of GA on DM-induced delayed wound healing in both keratinocytes and fibroblasts using high glucose-containing medium to mimic diabetes. In the current study, we found that GA has antioxidant properties, as well as cell migration effects in both normal and high glucose conditions, suggesting that GA has a curative potential for chronic wounds in DM.

## 2. Results

### 2.1. GA Protects Skin Cells from Oxidative Stress

**Figure 1:**
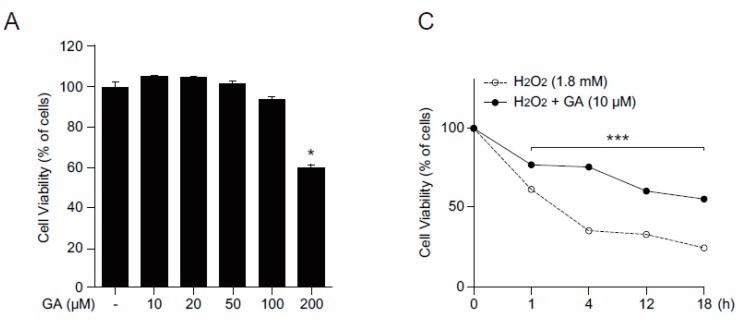

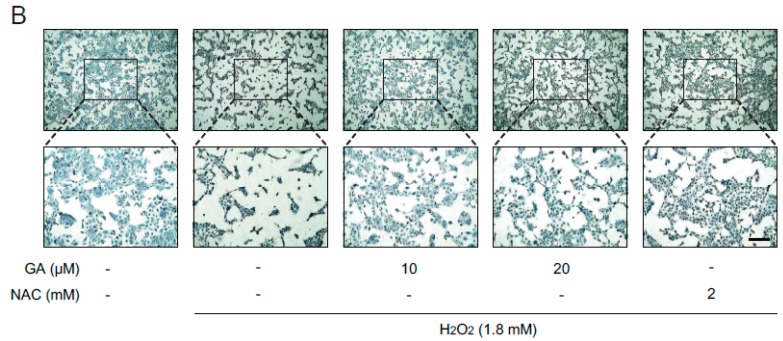
GA protects cells from oxidative stress in human keratinocytes. (**A**) HaCaT cells were treated with different concentrations of GA for 24 h and cell viability was assessed by MTT assay. The values represent mean ± SEM (* *p* < 0.05, one-way ANOVA followed by Bonferroni’s post hoc test); (**B**) HaCaT cells were pretreated with either vehicle or two different GA concentrations (10 and 20 μM) in the presence or absence of H_2_O_2_ (1.8 mM) and then incubated for 4 h. Cell viability was shown by methylene blue staining; and (**C**) cells were treated with vehicle or GA (10 μM) in the presence of H_2_O_2_ (1.8 mM) and cell viability was assessed in a time-dependent manner. The data are expressed as mean ± SEM (*** *p* < 0.05, two-way ANOVA followed by Bonferroni’s post hoc test). Scale bar = 200 μm.

Polyphenols are known to have a positive impact on cell viability by modulating oxidative stresses. To determine if GA has any effect on cell viability and oxidative stress, we first examined the cytotoxicity of GA in human keratinocytes, HaCaT cells using 3-(4,5-dimethylthiazol-2-yl)-2,5-diphenyltetrazoliumbromide (MTT) assay (Figure 1A). Although GA altered HaCaT cell viability at high concentration (200 μM), cell viability was not affected at lower concentrations (10, 20, or 50 μM). As such, low concentration of GA was used for subsequent experiments. MTT and methylene blue staining assays were performed to analyze cell viability following H_2_O_2_ treatment. *N*-acetylcysteine (NAC), a strong antioxidant, was used as an antioxidant control [14]. Pretreatment with GA and NAC significantly protected cells from H_2_O_2_-induced oxidative stress (Figure 1B) and protective effect of GA from ROS-induced cytotoxicity was observed at indicated time points (Figure 1C), demonstrating that GA protects cells from oxidative stress in human keratinocytes.

### 2.2. Antioxidant Effects of GA in Human Keratinocytes

**Figure 2:**
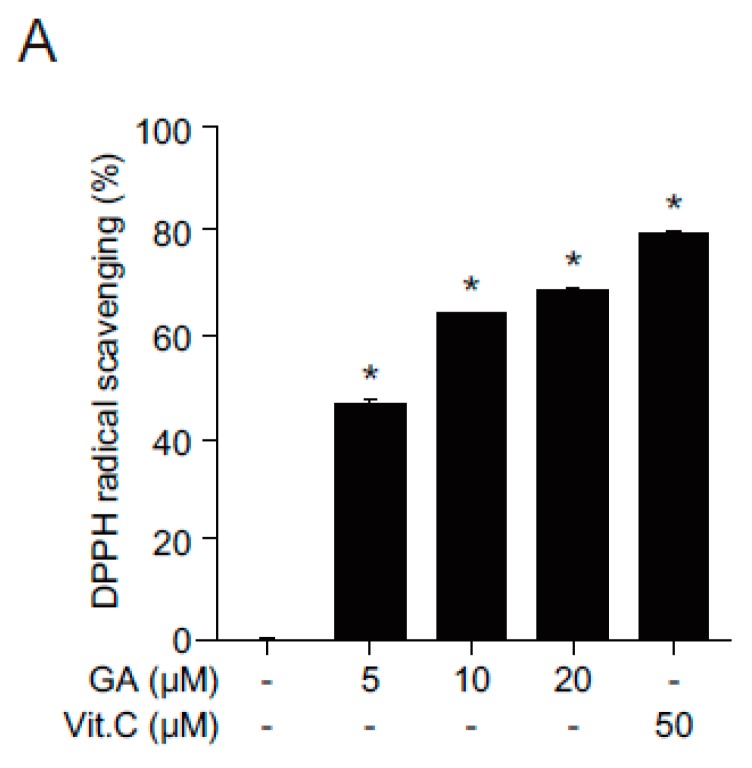

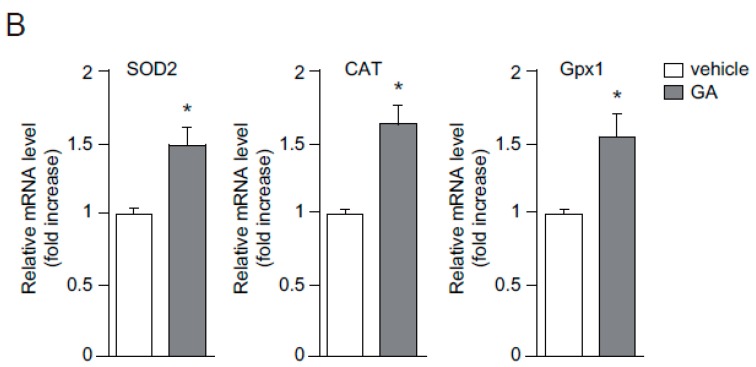
Antioxidant effects of GA in human keratinocytes. (**A**) Scavenging effects of GA on 2,2-diphenyl-1-picrylhydrazyl (DPPH) radical. HaCaT cells were treated with GA (5, 10, and 20 μM) or vitamin C (50 μM) for 30 min and assayed for antioxidant activity by DPPH scavenging assay. Vitamin C was used as a positive control for radical scavenging activity. Data are presented as the means ± SEM obtained from three independent experiments (* *p* < 0.05, one-way ANOVA followed by Bonferroni’s post hoc test); (**B**) HaCaT cells were incubated in the presence or absence of GA for 24 h and gene expression of SOD2, CAT, and Gpx1 was determined by quantitative real time-PCR (qPCR) method. 18S was used as a reference gene to measure relative gene expression. Values are expressed as mean ± SEM (* *p* < 0.05, Student’s *t*-test).

Free radicals are known to cause tissue injuries, hence contributing to the pathology of many human diseases [15]. To investigate the protective role of GA against oxidative stress in skin cells, the free radical scavenging activity of GA was examined. GA showed increased radical scavenging activity in a dose-dependent manner (Figure 2A). In addition, GA significantly upregulated the expression of antioxidant genes, including superoxide dismutase 2 (SOD2), catalase (CAT), and glutathione peroxidase 1 (Gpx1), in skin cells (Figure 2B). These results highly indicate that GA exerts its antioxidant function by directly upregulating the expression of these antioxidant genes.

### 2.3. GA Accelerates Wound Healing

**Figure 3:**
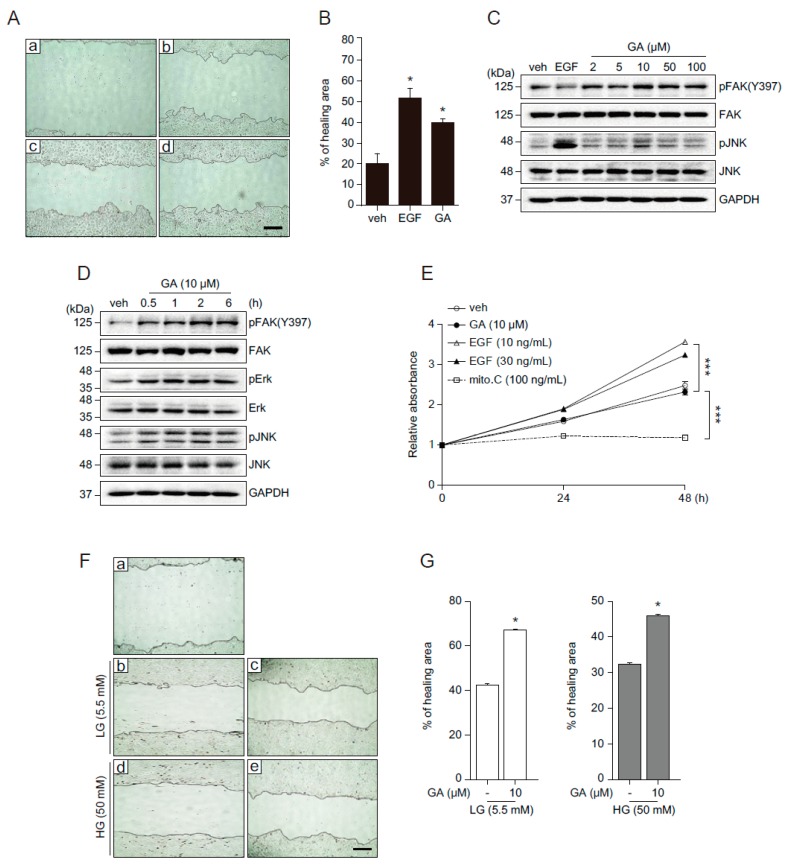
GA accelerates wound healing by activating cell migration in human keratinocytes. (**A**) Confluent monolayers of HaCaT cells were treated with vehicle (b), EGF (30 ng/mL, c), or GA (10 μM, d) after initial scratch (a) to induce a wound. Representative images of the wound in HaCaT cells were captured at the initial (time = 0, a) and 20 h later (b–d); (**B**) quantified percentage of healing area. Values are mean ± SEM obtained from three independent experiments (* *p* < 0.05, Student’s *t*-test); (**C**,**D**) HaCaT cells were treated with EGF (30 ng/mL), different concentrations of GA (2, 5, 10, 50, and 100 μM, C) or 10 μM of GA for indicated time periods (**D**) and subjected to Western blot with pErk, pJNK, pFAK, total form of Erk, JNK, and FAK, and glyceraldehyde 3-phosphate dehydrogenase (GAPDH) antibodies; (**E**) HaCaT cell growth following treatment of GA (10 μM) and EGF (10, 30 ng/mL) at indicated time points was analyzed by cell proliferation assay. Mitomycin C (100 ng/mL) was used as a negative control. Values are mean ± SEM obtained from three independent experiments (*** *p* < 0.05, Student’s *t*-test); (**F**) the effect of GA on cell migration in low glucose (LG, 5.5 mM) or high glucose (HG, 50 mM). Images were captured at initial (a) and at 20 h (b–e: b,d for vehicle, c,e for GA) after wounding. Mannitol was added for osmotic control; (**G**) percentage of wound healing area from the data shown in (**F**) was measured using Image J software (Version 1.48v, National Institute of Health, Maryland, USA). Data represent mean value ± SEM (* *p* < 0.05, Student’s *t*-test). Images were acquired using an AxioObserver FL microscope (Advanced Microscopy Group, Bothell, WA, USA) at ×10 magnification. Scale bar = 200 μm.

Although various phytochemicals have beneficial effect on skin physiology through regulation of redox homeostasis, the effect of GA on the skin physiology and pathophysiology remains unknown. To address this question, we examined the effect of GA on wound healing in human keratinocytes. HaCaT cells grown to confluence were wounded by scratching and then incubated in the presence of epidermal growth factor (EGF, well known for wound healing), GA, or vehicle for 20 h [16]. The image captured before the start of cell migration (right after wounding) indicates the initial gap in keratinocytes (Figure 3Aa). In the presence of vehicle, wound sites gradually closed (Figure 3Ab) and the rate of wound closure was significantly increased in EGF or GA treatment (Figure 3Ac,d), indicating that GA accelerated the wound closure in human keratinocytes (Figure 3B). The wound healing process involves cell proliferation and migration. Focal adhesion kinase (FAK), a cytoplasmic protein tyrosine kinase, responds to extracellular stimuli and regulates cellular processes such as proliferation and cell migration [17]. In *Drosophila* wing and abdomen wound models, c-Jun N-terminal kinase (JNK) is predominantly phosphorylated in the cells bordering the wound, indicating JNK signaling is required for epithelial cells at the wound edge to close the wound [18,19]. The mitogen-activated protein kinase (MAPK) signaling pathway also plays a role in the regulation of cell migration and wound healing [20]. To understand the molecular mechanism underlying GA-mediated wound healing and determine whether it is mediated through the activation of FAK and JNK, we examined FAK and JNK activation at different GA concentrations (2, 5, 10, 50, and 100 μM) (Figure 3C). GA strongly induced the phosphorylation of FAK and JNK with the highest activation peak observed at 10 μM. Next, we investigated the temporal regulation of GA (Figure 3D). Phosphorylation of FAK, extracellular signal-regulated kinases (Erk), and JNK was observed 30 min after treatment and maintained up to 6 h (Figure 3D). These results indicate that GA activates FAK, Erk, and JNK, which might be important for GA-mediated wound healing process. To determine whether GA-induced wound closure was mediated by proliferation or migration, we examined growth of HaCaT cells treated with EGF, GA, mitomycin C, or vehicle (Figure 3E). Mitomycin C, which blocks cell proliferation was used as a negative control [21]. Compared with vehicle treatment, EGF significantly increased cell proliferation, however, GA did not affect cell growth. This result suggests that GA might promote wound healing by increasing cell migration, not proliferation, in HaCaT cells.

### 2.4. GA Promotes Wound Healing Process in Hyperglucidic Condition

An increasing number of epidemiological investigations suggest that foods with high contents of phytochemicals and polyphenols are associated with lowering the risk of diabetes and its complications [22]. In recent years, polyphenols have been considered as appropriate nutraceuticals and supplementary applications for various aspects of DM due to beneficial effects on metabolism [23]. GA has an anti-metabolic syndrome activity, such as anti-hyperglycemic, antioxidant, and anti-lipidemic effects [24]. Moreover, we previously demonstrated the beneficial metabolic role of GA in high calorie diet [11]. One of the major characteristics of metabolic syndromes, including DM, is delayed wound repair. Based on GA’s beneficial effect on metabolism and wound healing, we sought to examine the effects of GA on wound healing in hyperglucidic conditions. A high glucose (HG) condition was used to mimic DM in a cell culture system [25,26,27,28]. We examined the wound healing effects at different glucose concentrations (5.5, 25, and 50 mM). Wound closure was delayed in cells incubated in high glucose conditions (25 and 50 mM) with significantly blunted wound closure at 50 mM compared to 25 mM glucose condition (data not shown). Therefore, 50 mM glucose condition was used as the hyperglucidic condition in subsequent experiments. HG delayed the rate of wound closure compared to LG condition (Figure 3Fa,b,d). GA significantly improved the rate of wound closure in the HG condition (Figure 3Fc,e and 3G), indicating that GA might be a good candidate for wound healing in hyperglucidic conditions.

**Figure 4:**
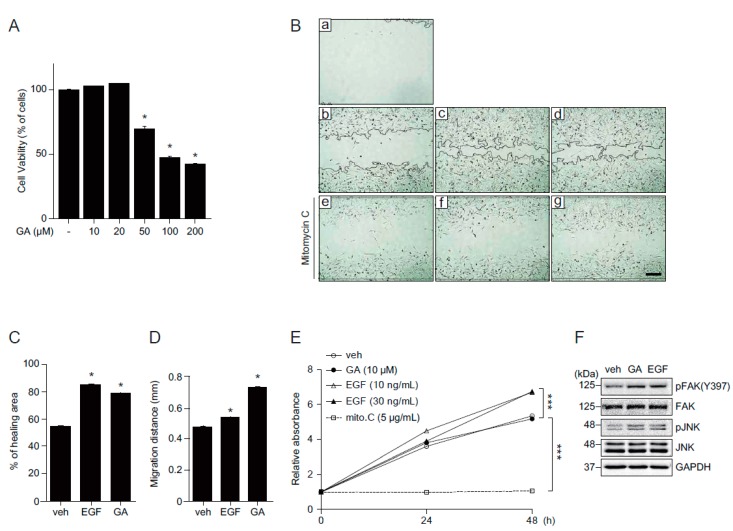
GA increases wound repair in mouse embryonic fibroblasts. (**A**) MEF cells were treated with different concentrations of GA (10, 20, 50, 100, and 200 μM) or vehicle for 24 h and subjected to cell viability using MTT assay. The values represent mean ± SEM (* *p* < 0.05, one-way ANOVA followed by Bonferroni’s post hoc test); (**B**) a scratch wound was performed on MEF cells. Cells were cultured with (e–g) or without (b–d) mitomycin C (5 μg/mL) and incubated for 12 h in the presence of EGF (c,f) or GA (d,g). Representative images were acquired at initial (time = 0, a) and at 12 h (b–g) after wounding; (**C**) percentage of healing area calculated from (**B**b–d); (**D**) migration distances in the presence of mitomycin C calculated from (**B**e–g); (**E**) MEF cell growth after GA or EGF treatment. Mitomycin C (5 μg/mL) was used as a negative control. Values are mean ± SEM obtained from three independent experiments (*** *p* < 0.05, Student’s *t*-test); and (**F**) activation of pFAK and pJNK in MEF cells treated with EGF (30 ng/mL) or GA (10 μM) for 30 min. Scale bar = 200 μm.

**Figure 5:**
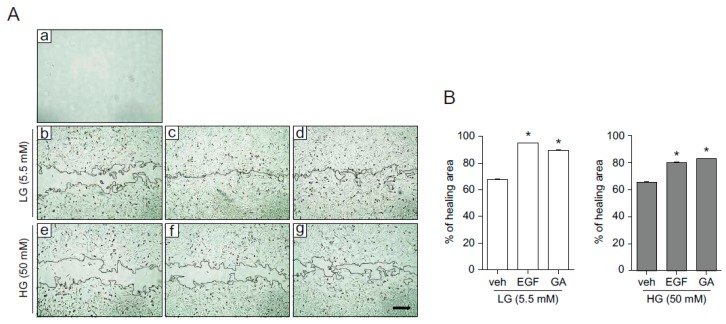

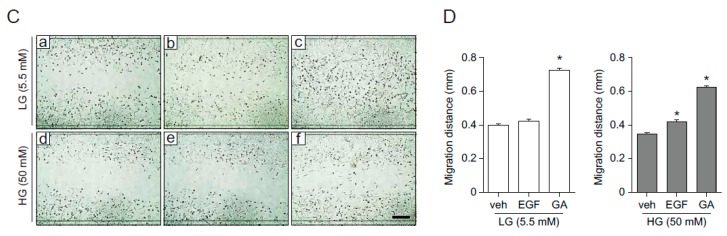
Effects of GA on wound healing in hyperglucidic condition. Mouse embryonic fibroblast cells were cultured in either LG (5.5 mM, **A**b–d, Ca–c) or HG (50 mM, **A**e–g, **C**d–f) conditions. (**A**) Wounded cells either in LG (b–d) or HG (e–g) conditions were treated with vehicle (b and e), EGF (30 ng/mL, c,f), or GA (10 μM, d and g) for 12 h. Mannitol was added for osmotic control. Images were captured at initial time (a) and at 12 h after scratching (b–g); (**B**) percentage of healing area for all groups from (**A**); (**C**) MEF cells either in LG (a–c) or HG (d–f) conditions were scratched and treated with vehicle (a,d), EGF (30 ng/mL, b,e), or GA (10 μM, c,f) for 12 h in the presence of mitomycin C (5 μg/mL); and (**D**) migration distance was measured from (**C**). Data represent mean value ± SEM obtained from three independent experiments (* *p* < 0.05, Student’s *t*-test). Scale bar = 200 μm.

Fibroblasts, within the dermis layer of the skin, play an important role in the skin wound healing process by generating the connective tissue required for wound repair. We, therefore, wondered if GA exerts its beneficial effects on fibroblasts as well. First, we examined the cytotoxicity of GA in mouse embryonic fibroblast (MEF) cells. MEF cells were treated with different concentrations of GA (10, 20, 50, 100, and 200 μM) for 24 h and cell viability was assessed by MTT assay. While high concentrations of GA (50, 100, and 200 μM) significantly decreased cell viability, relatively low concentrations of GA (10 and 20 μM) did not show observable cytotoxicity (Figure 4A). Consistent with the accelerated wound healing in keratinocytes, GA was also shown to stimulate wound repair in MEF cells (Figure 4Ba–d and 4C). To confirm the wound healing effect of GA resulted from increased cell migration, cells were pretreated with mitomycin C, a well-known proliferation inhibitor. Although GA showed slower wound closure rate compared to that of EGF (Figure 4Bc,d), cell migration in GA-treated cells was faster than EGF-treated cells in the presence of mitomycin C (Figure 4Be–g and 4D). To verify the effect of GA on MEF cell growth, proliferation assay was done in MEF cells (Figure 4E). While EGF stimulated cell growth, GA treatment did not induce any observable cell growth effects compared to vehicle treatment. Taken together, these results indicate that GA improves wound repair by increasing cell migration. Consistent with the keratinocytes, FAK and JNK were activated at 30 min after GA treatment in MEF cells, suggesting that the wound healing effect of GA might be commonly mediated through activation of FAK and JNK in both keratinocytes and fibroblasts (Figure 4F). To confirm whether GA also has a positive effect on wound repair in hyperglucidic condition in fibroblasts, we examined the wound healing effect of GA in fibroblasts in either LG or HG conditions (Figure 5). HG inhibited wound repair, as expected, and treatment with GA significantly improved wound healing similar to that of EGF (Figure 5A,B). Consistent with the wound healing mechanism of MEF cells in normal glucose conditions, the wound repair was significantly elevated in mitomycin C-treated cells in a hyperglucidic condition as well, indicating that the improved wound healing by GA in HG condition might not be due to cell proliferation but rather due to accelerated cell migration (Figure 5C,D). These results highly indicate that GA significantly improves wound healing not only in keratinocytes but also in fibroblasts.

### 2.5. The Effects of GA on Wound Healing in Human Fibroblasts

**Figure 6:**
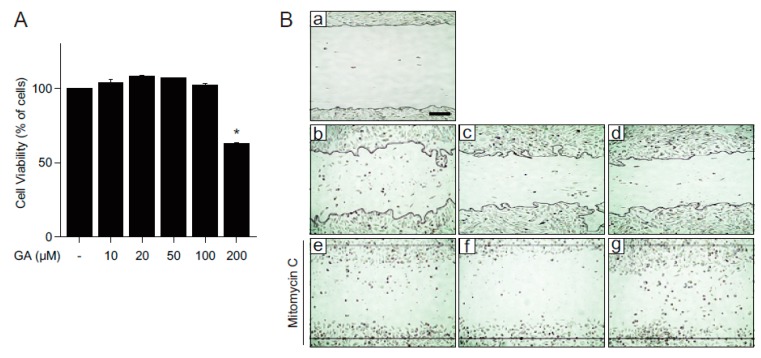

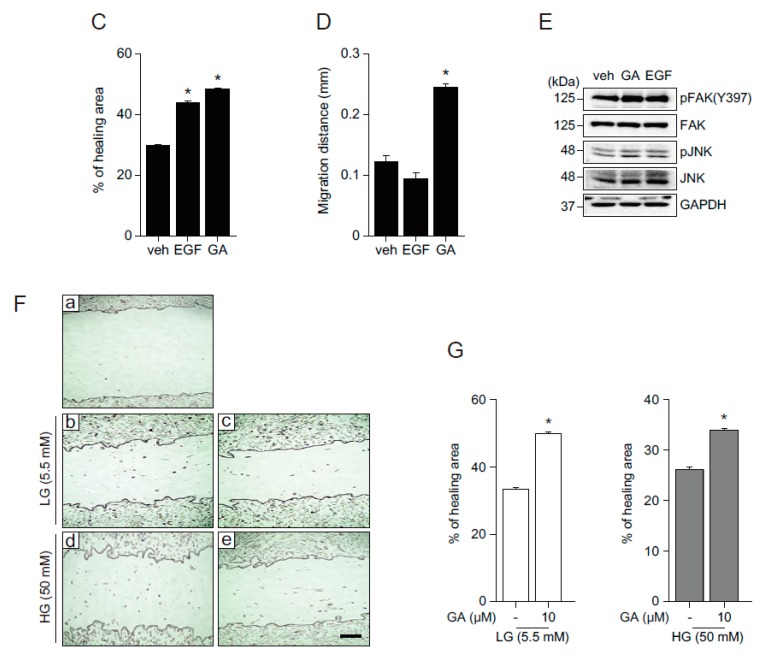
Effects of GA on wound healing in human fibroblast (HF21) cells. (**A**) HF21 cells were incubated in the absence or presence of GA (10, 20, 50, 100, and 200 μM) for 24 h and cell viability was assessed by MTT assay. The values represent mean ± SEM (* *p* < 0.05, one-way ANOVA followed by Bonferroni’s post hoc test); (**B**) in vitro wound healing assays in HF21 cells. Cells were treated with vehicle (b,e), EGF (30 ng/mL, c,f), GA (10 μM, d,g) after initial wounding and incubated in presence (e–g) or absence (b–d) of mitomycin C (5 μg/mL) for 20 h. Representative images were acquired at initial time (a) and 20 h (b–g) later. Percentage of healing area (**C**) and migration distances (**D**) were measured based on (**B**). Data represent mean value ± SEM (* *p* < 0.05, Student’s *t*-test); (**E**) activation of pFAK and pJNK in both GA (10 μM)- and EGF (30 ng/mL)-treated HF21 cells; (**F**) HF21 cells were cultured in either LG (5.5 mM) or HG (50 mM) conditions. Cells incubated in LG or HG were wounded by scratching and then incubated in the presence (c,e) or absence (b,d) of GA (10 μM) for 20 h; and (**G**) the percentage of healing area was measured from (**F**). Data represent mean value ± SEM (* *p* < 0.05, Student’s *t*-test). Images were acquired using AxioObserver FL microscope at ×10 magnification. Scale bar = 200 μm.

While mouse embryonic fibroblasts are known as a good model for embryonic mesenchymal tissue repair, we wanted to examine whether GA also has a beneficial effect on wound healing in human fibroblasts. To do this, we again examined the cytotoxicity of GA in human fibroblast, HF21 cells (Figure 6A). GA did not induce significant cytotoxicity in HF21 cells up to 100 μM. In addition, consistent with the results in keratinocytes and MEF cells, GA stimulated wound closure in HF21 cells significantly through increased cell migration (Figure 6B–D). Furthermore, the activation of FAK and JNK were also involved in wound healing in HF21 cells (Figure 6E). Next, to examine whether human fibroblasts behave the same as MEF cells on wound closure in hyperglucidic condition, in vitro wound healing assay was performed in HF21 cells in LG and HG conditions. In both LG and HG conditions, GA accelerated the wound closure, indicating that GA promotes wound healing in hyperglucidic condition in human fibroblasts (Figure 6F,G).

Collectively, the beneficial wound repair effects of GA in HG condition highly imply that GA might be a potential therapeutic agent for improving wound healing impairment resulting from metabolic syndromes, such as obesity and diabetes.

## 3. Discussion

Numerous biomedical studies have focused on polyphenols because epidemiological data indicate that polyphenol-rich diets have protective effects against chronic diseases, such as cancer and metabolic syndrome [29]. There are also continuously growing interests in beneficial effects of polyphenols on human skin: anti-inflammatory, anti-oxidant, anticancer, and anti-aging. Furthermore, several clinical trials and biomedical research have evaluated the effectiveness of novel polyphenolic therapies in remedy of dermatological diseases and conditions [29,30]. Among numerous polyphenols, gallic acid (GA) is found in almost all plants and is known to have powerful health benefits, including anti-diabetic properties [3]. Despite having a wide range of health benefits, the physiological roles of GA on the skin have not been identified. In this regard, the current study focused on the roles of GA in skin homeostasis and identified that GA accelerates wound healing by protecting skin cells from oxidative stress and by activating FAK, JNK, and Erk in human keratinocytes. More importantly, GA also significantly improved wound healing under a hyperglucidic condition by promoting cell migration (Figure 3, Figure 5, and Figure 6).

The human skin is continuously exposed to the harmful effects of sunlight, such as ultraviolet radiation (UVR) [31]. Continuous exposure to UVR accelerates DNA damage, inflammation, and oxidative stress. Oxidative stress by reactive oxygen species (ROS) is known as a key factor for skin alteration and skin aging [32]. Increased expression of antioxidant genes including catalase, SOD2, and Gpx1 by GA treatment suggests that GA might also have beneficial effect on skin aging. Thus, the protective effect of GA on skin aging, together with the underlying mechanism, might be another area for further study. Interestingly, GA also showed beneficial metabolic effect by activation of the AMPK/Sirt1/PGC1α pathway [11]. Since AMPK activation plays a role in redox homeostasis and increases gene expression of antioxidant enzymes, AMPK activation by GA might positively influence, at least in part, the antioxidant effect of GA [33]. 

Patients suffering from diabetes, obesity, and other metabolic syndromes manifest impaired wound healing [34,35]. In addition, hyperglycemia, a hallmark of diabetes, leads to increased oxidative stress and cellular damages, and induces detrimental effects on wound healing [36,37]. Based on beneficial effects of GA on oxidative and metabolic stresses, we investigated the effect of GA on wound repair under hyperglucidic conditions mimicking metabolic syndrome states. Our results showed that GA promotes wound healing under hyperglucidic conditions. In addition, increased FAK activity and the wound closure effect in the presence mitomycin C suggest that the beneficial effects of GA on wound healing might be mediated by enhancing cell migration in both epidermal keratinocytes and fibroblasts (Figure 3, Figure 4, and Figure 6). It has been reported that FAK overexpression enhances cell migration, whereas inhibition of FAK by either an inhibitor or KO model shows blunted cell migration [38,39,40]. Therefore, the activation of FAK by GA treatment in keratinocytes might be linked to the increased migration. Intriguingly, a recent study showed that hyperglycemia inhibits the activation of FAK, which might result in hyperglycemic-induced cell migration inhibition [37]. Therefore, the FAK activation by GA might play a role in enhancement of wound healing under hyperglucidic conditions. To verify the importance of FAK in wound healing by GA, especially under hyperglucidic conditions, further experiments using a specific inhibitor or knockdown of FAK would be necessary. In addition to the increased level of FAK, GA activates JNK and Erk, which are also involved in wound healing process [18,19,20]. Erk phosphorylation leads to the activation of transcription factors including Elk-1, which is implicated in the regulation of matrix metalloproteases (MMP)-2 and -9 and induce cell migration [41,42]. Therefore, in this regard, a further study examining whether Elk-1 and MMPs are regulated by GA through the activation of Erk might provide further molecular evidences on the effect of GA in wound healing.

In the skin, the epidermal keratinocytes layer is underpinned and connected to the dermis, which contains dermal fibroblasts as well as immune cells, blood vessels, and nerve fibers [43]. Fibroblasts contribute to the homeostasis of skin and physiopathological conditions including wounds, aging, and cancers in skin. During cutaneous wound healing, fibroblasts migrate, proliferate, and transform into cells involved in a number of key processes, such as breaking down fibrin clots, creating new extracellular matrix (ECM), and collagen structure to support other cells associated with effective wound healing. However, these effects are impaired in patients with metabolic syndrome, including diabetes and obesity, as well as in the elderly [44,45]. In line with those studies, our finding suggests that GA has a migratory effect on fibroblasts and that GA might be a potential therapeutic agent for treating both acute and chronic skin disorders, including wound healing impairment.

## 4. Materials and Methods

### 4.1. Cell Culture and Reagents

Human keratinocytes (HaCaT), mouse embryonic fibroblast (MEF), and human fibroblast (HF21) cells [46] were routinely cultured in Dulbecco’s modified Eagle’s medium (DMEM, Hyclone, Logan, UT, USA) containing 10% heat-inactivated fetal bovine serum (FBS) at 37 °C in 5% CO_2_. EGF, GA, NAC, H_2_O_2_, and mannitol were purchased from Sigma (St. Louis, MO, USA). Mitomycin C was purchased from Abcam (Cambridge, UK).

### 4.2. MTT Assay

Cell viability was determined using the 3-(4,5-dimethylthiazol-2-yl)-2,5-diphenyltetrazoliumbromide (MTT, Sigma-Aldrich) assay. Cells were cultured in 96-well microplates and incubated in media containing 10% FBS. 24 h later, cells were treated with the indicated doses of GA and subjected to MTT assay 24 h later. After treatment, the media was replaced with 100 μL of MTT solution (0.5 mg/mL in cell culture medium) and incubated at 37 °C for 4 h. MTT solution was then removed, and MTT formazan dissolved in 100 μL dimethyl sulfoxide (DMSO). Absorbance was measured at 540 nm using spectrophotometry. For time course cell viability following H_2_O_2_ treatment in HaCaT cells, cells were cultured in 96-well microplates. 24 h later, HaCaT cells were treated with H_2_O_2_ (1.8 mM) in the absence or presence of 10 μM GA, and subjected to MTT assay. The data are expressed as mean ± SEM of three independent experiments.

### 4.3. Methylene Blue Staining

Cells were cultured in six-well dishes for 24 h and then treated with NAC and different concentrations of GA in the absence or presence of H_2_O_2_. 4 h later, cells were washed twice with ice-cold PBS and fixed with 2 mL/six-well plate of ice-cold 95% (*v*/*v*) ethanol for 5 min. After fixation, cells were washed twice with PBS and stained with 2 mL ice-cold 2% methylene blue solution for 20 s. After staining, the cells were washed with distilled water and air dried. Images were captured under the microscope, AxioObserver FL microscope (Advanced Microscopy Group, Bothell, WA, USA) at ×10 magnification.

### 4.4. DPPH Free Radical Assay

2,2-diphenyl-1-picrylhydrazyl (DPPH) is a stable free radical at 515 nm and a trap (scavenger) for other radicals. Neutralization of the DPPH radical by an antioxidant compound leads to the disappearance of absorbance at 515 nm and this reduction in absorbance is used as a measurement of radical scavenging of the antioxidant compound [47,48]. DPPH and vitamin C were purchased from Sigma. DPPH stock solutions were prepared in ethanol and diluted to 0.1 mM final concentration. 0.1 mM DPPH solution was added to 0.5 mL of each sample and incubated at 4 °C for 30 min. After the reaction was complete, spectrophotometric measurements were taken at 517 nm using a Multiskan Go Spectrophotometer (Thermo Fisher Scientific, Waltham, MA, USA).

### 4.5. In Vitro Wound Healing and Migration Assays

Cells were cultured in six-well dishes and grown to confluence at 37 °C. A scratch wound was created by manually scraping the cell monolayer with a P100 pipet tip [49]. Cells were washed once and incubated with 2 mL of growth media containing EGF, GA, or vehicle. For migration assay, cells were wounded by scratching using a P100 pipet tip and treated with mitomycin C. Images were acquired at the indicated times using an AxioObserver FL microscope at 10× magnification. The width of the wound and migration distances were measured in more than three locations, and the percentage of wound healing area and migration distances were calculated using ImageJ software. Each experiment was performed in triplicate.

### 4.6. Western Blot Analysis

Cells were washed once with PBS and lysed with RIPA buffer (150 mM NaCl, 50 nM Tris, 1% Triton-X-100, 0.5% sodium deoxycholate, and 0.1% SDS) containing protease and phosphatase inhibitors (Roche, Indianapolis, IN, USA). Cell lysates were centrifuged at 12,000 *g* for 5 min to isolate protein extracts. The protein concentration was measured using Bio-Rad Protein Assay reagent (Bio-Rad Laboratories, Hercules, CA, USA). The same amount of protein was loaded and separated by electrophoresis on SDS-polyacrylamide gels, and then transferred to nitrocellulose membranes. The membranes were blocked in 5% skim milk in Tris-buffered saline (TBS) containing 0.1% Tween 20 for 1 h at room temperature. The blocked membranes were then incubated with primary antibodies overnight at 4 °C with agitation followed by incubation with horseradish peroxidase-conjugated secondary antibodies for 1 h at room temperature. The blots were visualized using the Chemiluminescence Western Blot Detection System (BioSpectrum^®^600 Imaging System, Upland, CA, USA). Primary antibodies used are as follows: pErk, Erk, pJNK, JNK, pFAK (Y397), and FAK from Cell Signaling (Danvers, MA, USA), and GAPDH from Santa Cruz Biotechnology (Santa Cruz, CA, USA).

### 4.7. Quantitative Real-Time PCR

Total RNA was isolated from cells using TRIzol reagent according to the manufacturer’s protocol (Invitrogen, Carlsbad, CA, USA). For RT-PCR, cDNAs were synthesized using a High Capacity cDNA Reverse Transcription Kit (Applied Biosystems, Foster City, CA, USA) and then amplified by PCR with SOD2, CAT, and Gpx1 primers: SOD2 (F; 5′-AATCAGGATCCACTGCAAGGA-3′, R; 5′-AGGCGTGCTCCCACACATC-3′), CAT (F; 5′-CTGAGTCTCTGCATCAGGTTT-3′, R; 5′-TCATGTGGCGATGTCCAT-3′), Gpx1 (F; 5′-GCACCCTCTCTTCGCCTTC-3′, R; 5′-TCAGGCTCGATGTCAATGGTC-3′). Real-time PCR was performed in triplicate using the 7900HT Fast Real-Time PCR System (Applied Biosystems). 18S was used as the control gene for normalization.

### 4.8. Cell Proliferation Assays

Cells were cultured in 96-well microplates for 24 h at 37 °C. Cells were washed and incubated with 100 μL of 0.5% serum media containing EGF, GA, mitomycin C, or vehicle. Assays were performed by adding 20 μL of the CellTiter96^®^ AQ_ueous_ One Solution Reagent (Promega Corporation, Madison, WI, USA) into culture wells and incubated at 37 °C for 4 h. The quantity of formazan product was measured at 490 nm absorbance using a 96-well plate reader. The data are expressed as mean ± SEM of three independent experiments.
